# Genetic risk for high body mass index before and amidst the obesity epidemic: Cross-cohort analysis of four british birth cohort studies

**DOI:** 10.1371/journal.pgen.1012138

**Published:** 2026-06-18

**Authors:** Liam Wright, Neil M. Davies, Gemma Shireby, Dylan M. Williams, Tim T. Morris, David Bann

**Affiliations:** 1 Centre for Longitudinal Studies, University College London, London, United Kingdom; 2 Division of Psychiatry, University College London, London, United Kingdom; 3 Department of Statistical Science, University College London, London, United Kingdom; 4 Department of Public Health and Nursing, Norwegian University of Science and Technology, Trondheim, Norway; 5 Unit for Lifelong Health & Ageing, University College London, London, United Kingdom; Newcastle University, UNITED KINGDOM OF GREAT BRITAIN AND NORTHERN IRELAND

## Abstract

Obesity is a highly heritable trait, but rising obesity rates suggest environmental change is also of profound importance. We conducted a cross-cohort analysis to examine how associations between genetic risk for high BMI and observed BMI differed in four British birth cohorts born before and amidst the obesity epidemic (1946, 1958, 1970 and ~2001; N = 19,379). BMI (kg/m^2^) was measured at multiple time points between ages 3 and 69 years. We used polygenic indices (PGI) derived from GWAS of adulthood and childhood BMI, respectively, with mixed effects models used to estimate associations with mean BMI and quantile regression used to assess associations across the distribution of BMI. We further used linear regression to estimate PGI-heritability (PGI-h^2^; incremental variance explained by the PGI) and Genomic Relatedness Restricted Maximum Likelihood (GREML) to calculate SNP-heritability (SNP-h^2^) by cohort and age. Adulthood BMI PGI was associated with BMI in all cohorts and ages but was more strongly associated with BMI in more recently born generations, e.g., at age 16y, a 1 SD increase in the adulthood PGI was associated with 0.46 kg/m^2^ (0.37, 0.55) higher BMI in the 1946c and 0.90 kg/m^2^ (0.83, 0.97) higher BMI in the 2001c. Cross-cohort differences widened with age and were larger at the upper end of the BMI distribution, indicating disproportionate increases in obesity in more recent generations for those with higher PGIs. Differences were also observed when using the childhood PGI. There were no clear, consistent differences in PGI-h^2^ or SNP-h^2^, possibly due to limited statistical power, except that PGI-h^2^ was highest in the most recently born cohort (2001c) when using the most predictive PGI for adulthood BMI. Findings highlight how the environment can modify genetic associations; genetic associations with BMI differed by birth cohort, age, and outcome centile.

## Author summary

Over the past five decades, obesity rates have risen sharply among both children and adults. While much research has explored the causes of this obesity epidemic, increases in body mass index (BMI) have not been uniform: levels of (extreme) obesity have grown faster than median BMI. This suggests that some individuals are more susceptible to aspects of the obesogenic environment than others. One possible reason for this is genetics, as BMI is a heritable trait. In this study, we used data from four British birth cohorts (born in 1946, 1958, 1970, and 2001, respectively) to examine whether the relationship between polygenic indices (PGIs) for BMI and measured BMI has changed across generations. We found evidence that genetic associations with BMI were progressively stronger in more recent cohorts, especially at older ages and among individuals with higher BMI. These findings suggest that people with a genetic predisposition to higher BMI are more susceptible to the obesogenic environment.

## Introduction

Obesity is a multifactorial disease characterized by excess adiposity and its sequalae [1]. Obesity is typically measured as (though not synonymous with) having a body mass index (BMI) exceeding 30 kg/m^2^. Obesity is a leading cause of morbidity and premature mortality worldwide [2], with the global economic cost of overweight and obesity estimated to exceed $2 trillion per annum [3]. More than one in four adults and one in five 11-year-olds in England is obese [4]. The strong tracking of BMI across the life course [5] raises the possibility that current generations will spend more time obese [6], increasing the risk of public health problems in years to come [7].

It was not always thus. The prevalence of obesity has increased dramatically in industrialised nations over the past five decades, though the timing and extent of this increase has differed markedly across countries [8]. In England, obesity rates among children and adults have more than tripled since the mid-1970s [9–12]. The precipitous increase in obesity, faster than any plausible genetic change at a population level, suggests an important role of the environment in determining body weight.

Obesity is likely proximally caused by an imbalance between energy consumed and energy expended. Multiple societal changes have occurred alongside the obesity epidemic that are thought to have negatively influenced energy balance, though the relative contribution of changes in energy consumption and expenditure remains debated [11,13–16]. Technological, economic and social developments have progressively ‘engineered’ physical effort out of many people’s lives [17], for instance, through the introduction of labour-saving devices in the home and in the workplace, the growth of sedentary leisure activities, such as watching television and playing video games, and the decline in manual employment [18]. Food, especially sugary and fatty food, has meanwhile become cheaper [17], the relative share of food expenditure on processed foodstuffs has increased [19], and fast-food outlets have expanded in number [20].

Yet, while obesity rates have increased, the underlying change in the population distribution of body mass index (BMI) has not been uniform. Instead, the distribution of BMI has become more variable and more skewed – levels of underweight are almost unchanged, while the increase in median BMI has been small relative to the growth in obesity rates [6,9,10,21]. This change in the distribution of BMI suggests that individuals differ in their susceptibility to the obesogenic environment. One source of these differences may be genetics. BMI is highly heritable, with estimates from twin studies ranging 47–90% [22]. Genetic variants that increase the risk of obesity may operate through the environment [23]. For instance, variants of the *FTO* gene strongly related to obesity risk [24] are also associated with multiple behavioural and psychological dispositions related to eating, such as increased hunger and lower satiety [25], including when adjusting for body size [26]. These dispositions may be more likely to translate into higher BMI in conditions where energy-dense food is cheap, salient, and widely available and where individuals are unlikely to compensate by increasing physical activity – hallmarks of the obesity epidemic [23,27].

Several gene-environment interaction (GxE) studies have investigated whether heritability and *genotypic associations* – defined as the association between genotype (e.g., operationalized as a polygenic index [PGI] or single genetic variant, such as FTO) and the absolute level of a trait (in this case, units [kg/m^2^] of BMI) – have increased alongside the obesity epidemic. These proxy for exposure to the obesity epidemic by birth year or year of assessment [28–36] or prevalence of obesity or mean BMI in the sample studied [37,38]. These studies show that, though average BMI has increased regardless of genotype [28], genotypic associations have increased in magnitude alongside the obesity epidemic, while heritability has stayed relatively stable; between-person differences in BMI according to genotype are now increased, but the *proportion* of variation in BMI explained is largely unchanged.

However, a limitation of existing studies is that they have almost exclusively used data from adults – particularly older adults from the US – rather than children or adolescents. This is important as genetic effects are distinct or have varying magnitude at different developmental stages [39–42]; two PGIs trained on adult and childhood BMI, respectively, are only correlated at r ~ 0.4 [43]. Further, the environments people encounter that are relevant for obesity change as individuals develop in ways that could influence genetic effects. For instance, young children are generally given less agency over the food they consume and have very different physical activity levels than adults. It is therefore unclear to what extent previous results generalise to younger age groups. Further, most use regional rather than national samples.

Previous studies have also focused on changes in the association of genetics and mean BMI or obesity, specifically, rather than investigating changing associations across the full distribution of BMI – as noted, the obesity epidemic is marked by increasing skewness in BMI [9,21]. Previous research has shown that an adult BMI PGI is particularly associated with high levels of BMI (e.g., Class II obesity) [43], but this has not been examined comparing cohorts differentially exposed to the obesity epidemic.

The British Birth Cohort Studies [44], which follow cohorts of individuals born in 1946, 1958, 1970, and 2000/02, offer a unique window into changing obesity rates. The cohorts have recently been genotyped and have multiple measurements of BMI across life, collectively spanning pre- and post-obesity epidemic periods. The oldest cohort grew up in a relatively uniform food environment and were eight years old when post-World War II food rationing ended in the UK [44,45]. In contrast, the youngest cohort had obesity rates of 20% by age 11y, four times the rate of similarly aged children twenty years prior [45].

Thus, in this study, we used data from the four British birth cohort studies to investigate how genetic associations with childhood and adulthood BMI have changed over the obesity epidemic in the UK. We compared genotype-phenotype associations for two PGI derived from GWAS of adulthood and childhood BMI, respectively, as well as using genome-wide (i.e., SNP-heritability) and single gene (e.g., FTO) approaches. We further examined whether changes in the magnitudes of genetic effects have occurred across the entire distribution of population BMI. We hypothesised that changes in genetic associations would track cross-cohort differences in BMI: genetic effects would be stronger, at a given age, in each successive cohort, driven by stronger associations at upper centiles of the BMI distribution.

## Materials and methods

### Ethics statement

Each cohort has received ethical approval for collection and analysis of genetic data from cohort members. Ethical approval was obtained from the National Health Service (NHS) Central Manchester Research Ethics Committee (07/H1008/168) for the Medical Research Council (MRC) National Survey of Health and Development, from the NHS South East Multi-centre Research Ethics Committee (01/1/44) for the National Child Development Study, from the NHS London - Brighton & Sussex Research Ethics Committee (15/LO/1446) for the British Cohort Study, and from the NHS London-Central Research Ethics Committee (13/LO/1786) for the Millennium Cohort Study. Each cohort obtained written consent from cohort members.

### Participants

The MRC National Survey of Health and Development (hereafter, 1946c) follows a sample of individuals born in mainland Britain (England, Scotland, or Wales) during a single week of March 1946. Cohort members were recruited by sampling singleton births, with babies with a parent employed in a non-manual occupation oversampled. The National Child Development Study (hereafter, 1958c) and the British Cohort Study (hereafter, 1970c) track all individuals born in mainland Britain in single weeks of March 1958 and April 1970, respectively. Immigrants to the UK born in these weeks were later added using school enrolment information. The Millennium Cohort Study (hereafter, 2001c) follows a sample of individuals from across the UK born between 2000/02. Participants were recruited using a two-stage stratified sampling design and sampled from selected postcode areas. Individuals from Northern Ireland, Scotland and Wales, ethnic minority backgrounds, or disadvantaged areas were oversampled. Given differences in cohort eligibility and increasing ethnic diversity within the UK, we restricted our analysis in each cohort to singletons of European ancestry born in England, Scotland, or Wales. European ancestry was determined genetically comparing cohort members against the 1000 Genomes European sample; for further detail, see Supporting Information in S1 File.

The 1946c, 1958c and 1970c were genotyped using whole blood samples collected at ages 53y, 44y, and 46y, respectively, while the 2001c were genotyped using saliva samples collected at 14y. The procedures used to genotype participants, as well as steps used to quality control (QC) and impute genetic data, are described further in the Supporting Information in S1 File. Procedures differed between each cohort but were identical where possible. 2,731 (50.9%) eligible participants in the 1946c had valid genetic data. Corresponding figures were 5,989 (37.0%) for the 1958c, 5,170 (31.5%) for the 1970c and 5,489 (41.1%) for the 2001c. Further detail on each study is available in cohort profiles [46–51].

### Measures

#### Body mass index.

We used BMI as our measure of adiposity given its availability and repeated measurement in each of the cohorts used here. Height and weight were obtained from participants at the following ages:

- 1946c: ages 4y, 6y, 7y, 11y, 15y, 20y, 26y, 36y, 43y, 53y, 63y, and 69y
- 1958c: ages 7y, 11y, 16y, 23y, 33y, 42y, 44y, 50y, and 55y
- 1970c: ages 10y, 16y, 26y, 29y, 34y, 42y, and 46y
- 2001c: ages 3y, 5y, 7y, 11y, 14y and 17y

Height and weight were collected via direct measurement by interviewers, health visitors, doctors, or nurses except in the following sweeps where self-report was used: ages 20y and 26y in the 1946c; ages 23y, 42y, 50y and 55y in the 1958c; and ages 26y to 42y in the 1970c. Self-report was additionally used in a small number of cases where it was not possible to obtain a valid measurement from participants (e.g., where the participant refused).

We converted height and weight to BMI using the standard formula (kg/m^2^). From age 20 + , we used previous or succeeding measurements of adult height, where missing. To remove the influence of implausible values, we excluded values for adult BMI outside the range 15–50 kg/m^2^ and for child BMI excluded values where age- and sex-adjusted BMI z-scores were beyond +/- 3 SD of the sample mean (calculated in each cohort follow-up, separately).

#### Polygenic indices for body mass index.

In primary analyses, we used two polygenic indices (PGIs) for adult BMI and child BMI, respectively, derived from genome-wide association studies (GWAS) of UK Biobank (UKB) data, a sample of approximately 500,000 British adults aged 39–73 years old at recruitment in 2006–2010 [52]; the use of UKB avoided clear sample overlap with our data, which other large GWAS [e.g., 53–55] did not. Adulthood BMI was measured objectively at baseline assessment in UKB [56], while childhood weight was captured by retrospective self-report with participants asked whether at age ten whether they were “thinner, plumper, or about average” relative to others [57]. Previous work using the 1946c shows this PGI shows a similar relationship to phenotypic BMI as a PGI derived from a GWAS of prospectively measured child-adolescent BMI, but which used the 1958c in its discovery sample and so could not be used here [53].

We calculated each PGI using PRSice-2 [58] limiting to clumped genome-wide significant hits (p < 5e-8, R^2^ < 0.01, 1,000 kb window) and disregarding ambiguous alleles, assuming additive genetic effects and, for comparability between cohorts, subsetting to single nucleotide polymorphisms (SNPs) genotyped or imputed (INFO > 0.8) in each cohort. For interpretability, we standardised the PGIs to have a mean of zero and a standard deviation of one across the combined sample. The final PGI scores were based on 505 (adulthood BMI) and 227 (childhood BMI) SNPs, respectively.

We assessed the robustness of our results repeating analysis with three alternative genetic measures. First, we used a PGI derived from the largest GWAS to date [55]. This overlapped with two cohorts used here (1946c and 1958c), but was significantly larger (5.1 million individuals, ~ 0.2% sample overlap) and predictive of BMI (R^2^ of 17.6% in UKB) than the UKB-specific GWAS [56]. Second, we alternatively used a PGI for adult fat mass percentage, specifically, again based on a GWAS of UKB data [56; 461 SNPs]: BMI PGI have previously been shown to be related to other measures of adiposity, such as body fat percentage [59], but BMI does not distinguish between fat and lean mass and height has increased over time [60] Third, we used a variable capturing the count of effect alleles for a variant within the FTO gene (rs1558902) that is related to fat mass and eating behaviour [61,62].

#### Covariates and auxiliary variables.

We included several variables as covariates. Depending on the model, these were: cohort member’s sex, verbal reasoning ability at age 10/11, maternal age at birth, mother’s years of education, family socioeconomic class, mother’s BMI, and cohort member’s first ten genetic principal components (PCs). Further detail on these variables is provided in the Supporting Information in S1 File.

### Statistical analysis

To investigate changes in polygenic associations with (mean) BMI across cohorts, we regressed BMI values at each age upon the PGI, repeated separately for each PGI and cohort. As BMI was measured repeatedly, we used mixed effects modelling, with person-specific random intercepts added to models (observations nested within individuals). Given previous evidence that associations between PGI and BMI vary non-linearly over the life course [43], we interacted the PGI and age, with age modelled with two natural splines [63]. In our primary analysis, we adjusted for sex, a dummy variable for BMI measurement type (direct or self-report), and first ten genetic principal components (PCs), the latter to account for population stratification [64]. From these regressions, we calculated marginal effects across the range of follow-ups in a given cohort (i.e., ages 3y, 4y, …, 16y, 17y in the 2001c) and then, for a given age in a pair of cohorts, calculated z-scores for differences in these marginal effects. As a sensitivity analysis, we also repeated each analysis, including additional adjustment for (a) family socioeconomic class, mother’s years of education and childhood cognitive ability and (b) mother’s age and BMI. Each of these were regarded as background factors that may explain changing associations.

To examine changes in the polygenic associations with the distribution of BMI across cohorts, we used quantile regression, estimating separate quantile regressions for each decile (10^th^, 20^th^, …, 90^th^ centiles) of BMI (kg/m^2^), repeated for each PGI, age of follow-up, and cohort, and adjusting for sex, self-report dummy, (linear) age and the first ten genetic PCs. Estimates were then plotted and visually compared for each cohort, age, and outcome decile.

The above analyses examined cohort-by-PGI interactions on the absolute (i.e., kg/m^2^) scale. Since interaction results may differ on the absolute or relative scale, we performed three separate analyses to explore changes in the relative contribution of genetics to BMI across cohorts. First, we estimated ‘PGI-heritability’ (PGI-h^2^) by calculating incremental variance explained by each PGI. This was calculated by extracting R^2^ values from OLS regressions of BMI upon the PGI plus covariates (age, sex, and 10 PCs) and comparing these against R^2^ values obtained when not adjusting for the PGI. We repeated this for each PGI, cohort, and follow-up age separately and estimated 95% confidence intervals using bootstrapping (500 bootstraps, percentile method). Second, we transformed BMI into age- and cohort- specific ranks and projected these ranks onto the normal distribution using the inverse-normal quantile transformation. We then ran linear regressions with this alternate outcome measure for each PGI, cohort and follow-up age separately. This allowed us to determine whether PGI were differentially related to BMI *rank* within each cohort across generations.

Third, we estimated SNP-heritability (SNP-h^2^) at each follow-up using Genomic Relatedness Restricted Maximum Likelihood (GREML), as implemented in the software GCTA [65]. This method exploits variation in genetic relatedness between sampled, not closely related, individuals to calculate the proportion of phenotypic variance that can be explained (additively) by measured genetic variants [66]. The benefit of this approach is it does not rely on PGI weights, which here were based on the GWAS of an older population (i.e., UKB) and could, if causal genetic signals differ by cohort, be biased for younger cohorts.

We carried out all regression analyses using R version 4.3.1 [67]. Given the 1946c and 2001c used stratified study designs, we used study-specific probability (recruitment) weights in all analyses except for the GREML analysis, as the software did not allow for the inclusion of weights. We used (regression-specific) complete case data. Sample sizes therefore differed across analyses due to missing data for PGI, BMI or covariates, loss to follow-up, death, and emigration. In sensitivity analyses, we created bespoke inverse probability weights to account for selection into the genotyped sample and combined this with multiple imputation to address remaining item missingness. The procedure we used is described further in the Supporting Information in S1 File.

As a final sensitivity analysis, we repeated OLS regression models for childhood BMI (<19y) using age- and sex-adjusted BMI z-scores instead of raw BMI. This procedure projects raw BMI onto growth charts derived from a reference sample (in this case, UK children and adolescents between 1978–1990 [68]) and accounts for the rapid change in BMI during development.

## Results

### Descriptive statistics

Samples sizes were 2,731 (50.9% of eligible participants) for the 1946c, 5,989 (37.0%) for the 1958c, 5,170 (31.5%) for the 1970c and 5,489 (41.1%) for the 2001c. The number of eligible participants with BMI data at a given cohort-sweep is shown in Table A in S1 File.

**Fig 1:**
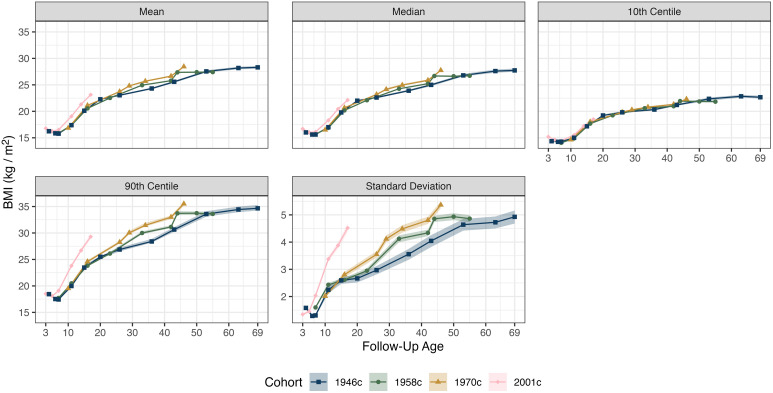
Descriptive statistics (+ 95% CIs) for raw BMI (kg/m^2^) by cohort and age at follow-up among genotyped participants. Estimates are weighted using recruitment weights and account for complex survey design.

The mean and variance of BMI were higher at a given age in each successive cohort, though differences between the 1946c, 1958c, and 1970c only arose during early/mid adulthood (Fig 1). The increase in variance was driven by increases at higher centiles of the BMI distribution – there was relatively little difference between cohorts in the prevalence of underweight or median BMI, while differences at the 90^th^ centile were substantially larger. Mean BMI increased as each cohort aged, but the rate of increase was greatest in the 2001c.

PGI distributions were similar in each cohort (Fig A in S1 File). Adulthood and childhood PGIs were positively correlated in each cohort; correlations ranged 0.34 – 0.36. The adulthood PGI was related to several covariates (Table 1), including positive correlations with mother’s (ρ = 0.08 – 0.14) and father’s BMI (ρ = 0.06 – 0.10) and negative correlations with participant verbal cognitive ability scores (ρ = -0.07 – -0.04). The adulthood PGI was also (negatively) related to parents’ education and maternal age. The childhood PGI was related to the mother’s and father’s BMI (ρ = 0.08 – 0.12 and 0.05 – 0.09, respectively; Table B in S1 File). Associations with other covariates, including verbal cognitive ability scores, were weaker in size and close to the null.

**Table 1:** Association between adulthood PGI and covariates.

	Variable	1946c	1958c	1970c	2001c
Sex	Male	-0.01 (1.01)	-0.02 (1.01)	0.01 (1.00)	0.00 (1.01)
	Female	0.00 (1.00)	-0.03 (0.98)	0.03 (0.99)	0.02 (1.01)
	Verbal Score @ Age 10/11	-0.04 (-0.09, 0.01)	-0.04* (-0.06, -0.01)	-0.04* (-0.07, -0.01)	-0.07* (-0.10, -0.04)
	Mother’s BMI	0.14* (0.09, 0.19)	0.09* (0.06, 0.12)	0.12* (0.09, 0.15)	0.08* (0.06, 0.11)
	Father’s BMI	0.06* (0.01, 0.12)	0.07* (0.04, 0.10)	0.09* (0.06, 0.13)	0.10* (0.07, 0.13)
	Mother’s Age	-0.06* (-0.11, -0.01)	-0.02 (-0.04, 0.01)	-0.01 (-0.04, 0.01)	-0.08* (-0.11, -0.05)
	Mother’s Education (Years)	-0.03 (-0.08, 0.02)	-0.01 (-0.04, 0.02)	-0.05* (-0.08, -0.02)	-0.10* (-0.13, -0.07)
	Father’s Education (Years)	-0.03 (-0.07, 0.01)	-0.04* (-0.07, -0.02)	-0.06* (-0.09, -0.03)	-0.08* (-0.12, -0.05)
Family Class (Registrar General)	I Professional	-0.31 (1.10)	-0.15 (1.03)	-0.14 (0.96)	-0.22 (1.00)
	II Intermediate	-0.06 (1.02)	-0.09 (0.99)	0.00 (1.00)	-0.03 (1.00)
	III Skilled Non-Manual	-0.11 (0.99)	-0.08 (0.98)	0.02 (1.01)	0.01 (1.00)
	III Skilled Manual	0.07 (1.00)	-0.01 (0.99)	0.07 (0.98)	0.14 (1.01)
	IV Semi-Skilled	-0.02 (0.96)	0.00 (1.00)	0.03 (1.02)	0.11 (1.02)
	V Unskilled	-0.01 (0.99)	0.16 (0.93)	0.05 (0.93)	0.01 (1.07)
	Not Working				0.16 (1.03)

For categorical covariates, cells show the mean (SD) of the adulthood PGI within a level of the covariate.

For continuous covariates, cells show the Pearson’s correlation coefficient (+ 95% CIs) between the adulthood PGI and the covariate.

‘*’ indicates the covariate is a statistically significant predictor of the PGI (Wald test, p < 0.05).

Estimates were weighted using recruitment weights and account for complex survey design. Row-wise complete case data.

Full definitions for each variable are provided in the Supporting Information in S1 File.

There was evidence of differential selection in the genotyped samples. In each cohort, compared with other cohort members, genotyped individuals were more likely to be from advantaged socioeconomic backgrounds (as measured by family socioeconomic class and parental education) and had higher cognitive ability, on average (Table C in S1 File). Higher BMI and PGI values were related to a greater likelihood of dropping out of a survey in later sweeps (Figs B and C in S1 File).

### Changing polygenic associations with (Mean) BMI

**Fig 2:**
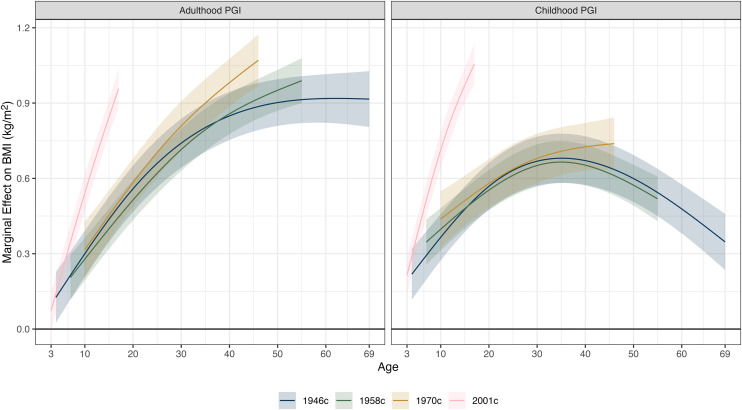
Association (+ 95% CIs) between PGI and BMI (kg/m^2^) by cohort, age, and PGI (adulthood or childhood). Derived from separate linear mixed effects models with the association between PGI and BMI allowed to vary by age (two natural splines). Adjustment was made for age (two natural splines), sex, first 10 genetic principal components, and a person-specific random intercept. Estimates were weighted using recruitment weights.

The adult PGI was positively associated with BMI in each cohort in childhood, adolescence and adulthood (left panel, Fig 2). Associations strengthened as participants aged. These associations were stronger in successively younger cohorts – especially the 2001c – but the age at which differences between cohorts appeared grew earlier over time (top panels, Fig D in S1 File). For instance, associations between the adulthood PGI and BMI were stronger in the 1958c than the 1946c only in mid-adulthood (~ age 40+). In comparison, differences between the 2001c and earlier cohorts appeared from childhood. At age 16y, a 1 SD increase in the PGI was associated with a 0.90 kg/m^2^ (0.83, 0.97) higher BMI in the 2001c, almost double the 0.46 kg/m^2^ (0.37, 0.55) difference estimated in the 1946c. Corresponding figures for the 1958c and 1970c were 0.42 kg/m^2^ (0.34, 0.50) and 0.48 kg/m^2^ (0.38, 0.58), respectively. At age 42y, a 1 SD increase in the PGI was associated with a higher BMI of 0.86 kg/m^2^ (0.77, 0.96) in the 1946c, 0.88 kg/m^2^ (0.80, 0.96) in the 1958c, and 1.01 kg/m^2^ (0.92, 1.11) in the 1970c. Note, these are of comparable size to the associations as early as age 16y in the 2001c (for contour plots, see Fig E in S1 File).

The childhood PGI was also positively associated with BMI in each cohort in childhood, adolescence and adulthood (right panel, Fig 2). Associations had an inverted-U shaped relationship with age, growing stronger into mid-adulthood but weaker thereafter. Associations were again considerably larger in the 2001c (bottom panels, Figs D and F in S1 File). However, there was little consistent difference between the 1946c and 1958c. At age 16y, a 1 SD increase in the PGI was associated with a higher BMI of 0.50 kg/m^2^ (0.41, 0.59) in the 1946c, 0.50 kg/m^2^ (0.42, 0.58) in the 1958c, 0.53 kg/m^2^ (0.43, 0.62) in the 1970c, and 1.01 kg/m^2^ (0.94, 1.09) in the 2001c.

### Changing polygenic associations with the distribution of BMI

**Fig 3:**
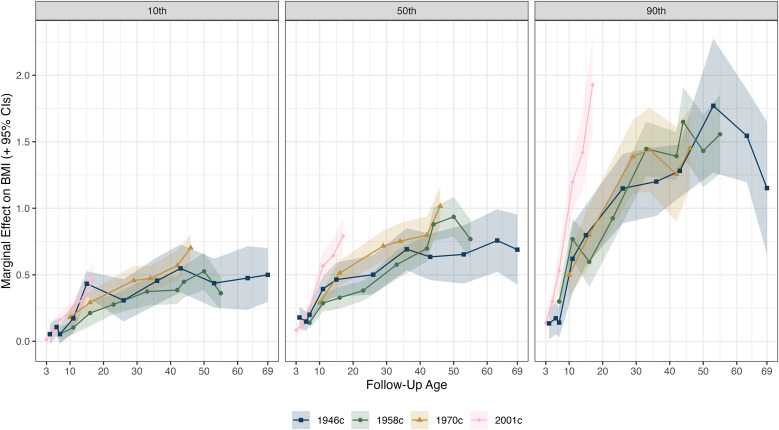
Association (+ 95% CIs) between adulthood PGI and BMI (kg/m^2^) by BMI decile, cohort, age of follow-up. Derived from separate quantile regressions adjusting for age (linear term), sex and first 10 genetic principal components. Estimates were weighted using recruitment weights. Each panel displays associations for a particular, selected decile of BMI (10^th^, 50^th^, 90^th^). Results show how the (conditional) centiles of BMI vary according to 1 SD increases in the adulthood PGI. Results displayed separately by cohort are displayed in Fig H in S1 File. Full results (10^th^, 20^th^, …, 90^th^ deciles) are displayed in Fig G in S1 File.

In quantile regression models, the association between the adulthood PGI and BMI was stronger at higher centiles of BMI in each cohort, indicating greater variance and skewness in BMI among those with higher PGI values (selected results shown in Fig 3; full results shown in Figs G-I in S1 File). Differences between the 2001c and the earlier cohorts in the association between the adulthood PGI and BMI were more pronounced at higher centiles of the distribution. At age 10/11, at the 10^th^ centile of the BMI distribution, a 1 SD increase in the adulthood PGI was associated with higher BMI of 0.17 kg/m^2^ (0.06, 0.28) in the 1946c, 0.10 kg/m^2^ (0.06, 0.15) in the 1958c, 0.18 kg/m^2^ (0.11, 0.25) in the 1970c, and 0.25 kg/m^2^ (0.18, 0.31) in the 2001c. Corresponding figures at the same age for the 90^th^ centile were 0.62 kg/m^2^ (0.36, 0.88) in the 1946c, 0.77 kg/m^2^ (0.61, 0.92) in the 1958c, 0.50 kg/m^2^ (0.34, 0.66) in the 1970c, and 1.20 kg/m^2^ (0.99, 1.40) in the 2001c, respectively. Differences in association in adulthood between the oldest three cohorts were less pronounced. Stronger associations at higher centiles, particularly for the 2001c, were generally also observed when examining the association between the childhood PGI and BMI scores (Figs I-K in S1 File).

### Changes in the relative contribution of genetics to BMI

**Fig 4:**
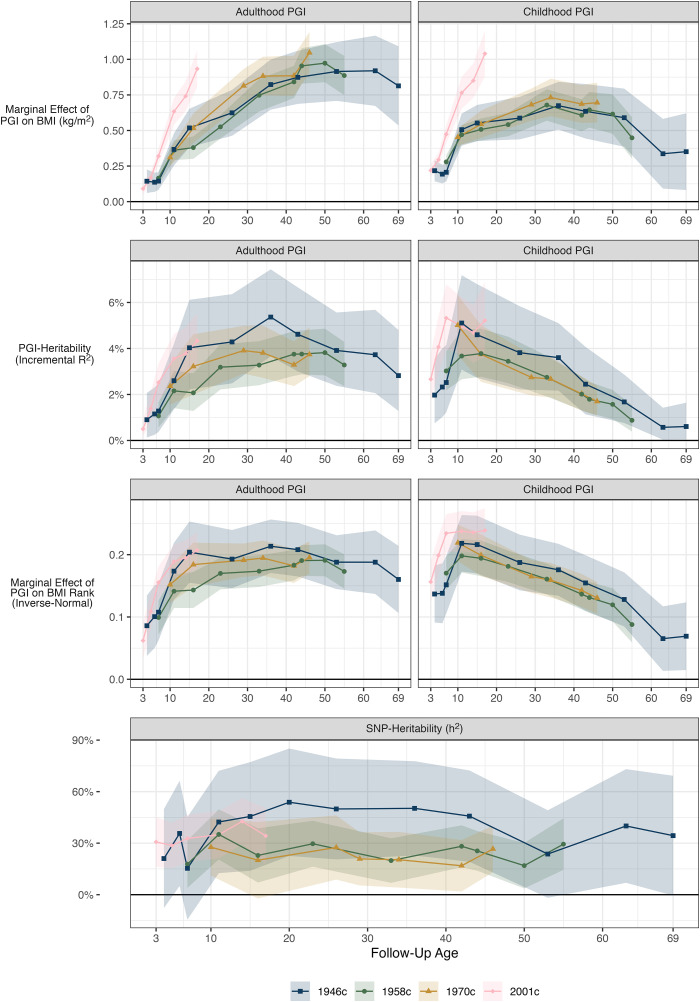
Relative contribution of genetics to BMI by cohort and follow-up. Top Panels: Marginal effect (+ 95% CIs) of 1 SD higher PGI on BMI (kg/m^2^), derived from regressions of BMI (kg/m^2^) upon PGI by cohort, age of follow-up, and PGI (adulthood or childhood). OLS regressions were performed separately for each cohort-sweep adjusting for age, sex and first 10 genetic principal components. Estimates were weighted using recruitment weights and account for complex survey design. Second Row: incremental proportion of variance explained (+ 95% CIs) by (adulthood or childhood) PGI (i.e., PGI-heritability) calculated by comparing R^2^ with regression of BMI on age, sex and first 10 genetic principal components, with and without further adjustment for the PGI. Confidence intervals estimated using bootstrapping (500 bootstraps, percentile method). Third Row: Marginal effect (+ 95% CIs) of 1 SD higher PGI on BMI (inverse-normal transformed) *rank,* derived from regressions of BMI ranks upon PGI by cohort, age of follow-up, and PGI (adulthood or childhood). Bottom Panel: SNP-heritability (+ 95% CIs) of BMI calculated with GCTA, adjusting for sex, age and first 10 genetic principal components. Survey weights were not incorporated in the GCTA analysis.

Though associations between the adulthood PGI and BMI increased in magnitude as participants aged (top left panel, Fig 4), the variance in BMI explained by the adulthood PGI (i.e., PGI-h^2^) stayed largely constant across adulthood (left panel, second row, Fig 4; also see Table D in S1 File) reflecting the higher variance in BMI at older ages. The adulthood PGI explained at most 5.4% of the variance in BMI in the 1946c (36y; 95% CI = 3.7%, 7.4%), 3.8% in the 1958c (50y; 95% CI = 2.8%, 4.9%), 3.9% in the 1970c (29y; 95% CI = 2.9%, 5.0%), and 4.3% in the 2001c (17y; 95% CI = 3.2%, 5.5%). There were no clear, consistent cohort differences in PGI-h^2^ using the adulthood PGI. Similarly, there were no clear consistent cohort differences in association between the adulthood PGI and BMI (inverse-normal transformed) *rank* (left panel, third row, Fig 4).

The proportion of variance in BMI explained by the childhood PGI increased into adolescence but declined thereafter (right panel, second row, Fig 4; also see Table D in S1 File). The childhood PGI explained at most 5.1% of the variance in BMI in the 1946c (11y; 95% CI = 3.1%, 7.2%), 3.8% in the 1958c (16y; 95% CI = 2.8%, 4.9%), 5.0% in the 1970c (10y; 95% CI = 3.7%, 6.3%), and 5.3% in the 2001c (7y; 95% CI = 4.0%, 6.8%) Again, there were no clear and consistent cohort differences in PGI-h^2^ using the childhood PGI, nor were there in the association between the childhood PGI and BMI (inverse-normal transformed) *rank* (right panel, third row, Fig 4).

SNP-h^2^ estimates estimated with GREML were larger than PGI-h^2^ estimates and ranged from 15.4% (7y) to 53.8% (20y) in the 1946c, 16.9% (50y) to 35.1% (11y) in the 1958c, 16.8% (42y) to 27.7% (10y) in the 1970c, and 28.6% (5y) to 42.7% (14y) in the 2001c (bottom panel, Fig 4; also see Table D in S1 File). SNP-h^2^ was greatest in the 1946c, but at each follow-up and in each cohort, confidence intervals were wide. Given the lack of precision in the estimates, there was no clear trend in SNP-h^2^ by age.

### Sensitivity analyses

Results for the associations between each PGI and mean BMI (kg/m^2^) were qualitatively similar when additionally controlling for family socioeconomic position and childhood cognitive ability or maternal BMI and maternal age at birth (Fig L in S1 File). Results were also qualitatively similar when using weighting to account for selection into the genotyped sample (Fig M in S1 File).

Cross-cohort differences in mixed-effects models were more pronounced when using the PGI from the large (> 5 million discovery sample) multi-ancestry GWAS, including clearer differences between the 1946c and 1958c (top right panel, Fig N in S1 File). Again, differences in association between cohorts in the 1946c, 1958c and 1970c only appeared during adulthood. At age 42y, a 1 SD higher multi-ancestry PGI was associated with higher BMI of 1.75 kg/m^2^ (1.67, 1.84) in the 1946c, 2.02 kg/m^2^ (1.95, 2.09) in the 1958c, and 2.21 kg/m^2^ (2.12, 2.30) in the 1970c. With the multi-ancestry PGI, there was also evidence that genetics had a greater relative contribution to BMI in the 2001c than earlier cohorts, either defined as PGI-h^2^ (middle right panel, Fig O in S1 File) or association between PGI and BMI *rank* (bottom right panel, Fig O in S1 File).

Mixed effects model results were partly replicated when using the PGI for adult fat mass rather than a PGI for adult BMI, specifically: the PGI had the strongest association in the 2001c (bottom left panel, Fig N in S1 File). This was also the case when using the rs1558902 *FTO* variant, though confidence intervals overlapped owing to the lower predictive power of the variable (bottom right panel, Fig N in S1 File). Results were qualitatively similar using age- and sex-adjusted BMI z-scores as the outcome variable rather than raw BMI: associations were strongest in the 2001c and the strength of the association between PGI and BMI increased from childhood to adolescence (Fig P in S1 File).

## Discussion

### Summary of results

Using multiple national birth cohorts with data spanning 1950–2018, we found considerable cross-cohort differences in associations of genetics with BMI. In each cohort, the magnitude of the association of the adulthood PGI with phenotypic BMI (kg/m^2^) increased as individuals aged, but the relationship was strongest in the most recent-born cohort (2001c). Differences in the strength of association of the adulthood PGI between the 1946c, 1958c and 1970c arose during adulthood, tracking the development of cross-cohort differences in BMI among these cohorts. Associations between the adulthood PGI and BMI were stronger at higher centiles of BMI in each cohort, consistent with genetic effects disproportionately impacting obesity rates. Cross-cohort differences in effects were also typically larger at higher centiles of the BMI distribution. Again, this tracked the nature of cross-cohort differences in BMI which were driven by increases in obesity, in particular. Cross-cohort differences in association between PGI and BMI were also observed using PGI for childhood BMI and adult fat mass and using results from a large (> 5 million participant) multi-ancestry GWAS.

However, cross-cohort differences in the relative contribution for phenotypic BMI were less consistent or clear. For both the childhood and adulthood PGI, the PGI-h^2^ (i.e., incremental variance explained by the PGI) were broadly similar in each cohort, as were the associations between the PGIs and BMI *rank*. Nevertheless, confidence intervals were wide reflecting low statistical power for the PGI; SNP-h^2^ appeared underpowered too. PGI-h^2^ was greater in the 2001c than the other cohorts when using the (substantially more predictive) multi-ancestry PGI.

### Explanation of results

The finding of larger associations between PGI and BMI in cohorts more affected by the obesity epidemic is consistent with previous studies in adults and twins [28–38,69]. We extend these results by examining data across life in multiple national cohorts and undertaking a range of analyses from distributional modelling to PGI, genome-wide, and specific gene approaches to inference. Changes in genetic association appear to have tracked the obesity epidemic – specifically, the timing at which differences in phenotypic BMI across cohorts has arisen and the disproportionate effects on obesity rates.

Why the adulthood and childhood PGIs have stronger associations with BMI in more recent born cohorts is unclear. While birth year is arguably an exogenous source of environmental variation, it does not distinguish which aspects of the environment have led to the changes observed. Previous studies have shown weaker effects of PGI on BMI among physically active individuals [70] or living in greater proximity to fast-food restaurants [71, though also see 72]. As the environment has changed, it may have enabled greater expression of genetic liability towards higher calorie consumption and, thus, higher BMI.

Factors that predict increasing strength of genetic association over time may also explain the stronger effects we observed at the upper centiles of the BMI distribution. In the 2001c, for example, proximity to fast food outlets varies considerably between cohort members and across time [73]. If genetic effects are of greater importance where calorific food is more readily available, we would, therefore, expect heterogeneous genetic effects. This would be reflected as greater variation and skewness in the distribution of BMI over time, as observed here. Moreover, some, but not all, individuals may offset greater genetic risk through higher physical activity or other changes in behaviour [70], which may again lead to greater variation in BMI among those with high PGI values.

Further work is required to identify the specific environmental factors responsible for PGI and BMI association heterogeneity. As has been argued, increasing associations between genes and adiposity over the obesity epidemic may not be specific but reflect increasing effects of all determinants of obesity [74,75]. However, we did observe higher PGI-h^2^ in the 2001c, compared with other cohorts, at least when using the multi-ancestry PGI.

Cohort differences may also be driven by genetically-influenced nurture. Recent work suggests that maternal BMI-related genetic variants that are not directly inherited influence offspring BMI [59,76,77]. Parents make choices on children’s behalf, and children may also model parent behaviour. Given changes in adult eating and exercise habits over time, this could explain some of the change in genetic effects in the 2001c relative to earlier cohorts. An extension of this work would be to investigate how much of the effects on BMI mean and variance are due to direct versus indirect genetic effects.

### Strengths and limitations

Strengths include using BMI data collected from nationally representative samples at multiple ages in each cohort, particularly measurements from early childhood and adolescence. BMI was also measured objectively on most measurement occasions and the cohorts we used spanned a wide period of recent history, including before the obesity epidemic in the UK. Data collection also overlapped with a period of post-war food rationing for the 1946c.

Limitations include the high degree of attrition (>50%) reflected in the genotyped samples. Individuals with higher BMI had higher rates of drop-out in each cohort, which may have biased results. Nevertheless, similar results were obtained when accounting for selection into the genotyped sample with inverse probability weighting. Our main analyses relied upon GWAS of an older cohort that did not span all age ranges or birth years used here. However, arguably this should bias towards finding larger effects in older cohorts, the opposite of what we found in our data. There are numerous pathways through which the PGI influence BMI and multiple changes marking increasing ‘obesogenicity’ of the environment, making interpretation difficult. Though, in sensitivity analysis, we used a variant in the FTO gene that has been shown to be related to several food-related behaviours and dispositions, it is challenging to identify the specific mechanisms underpinning links between genetic variants and higher weight (e.g., since higher weight may lead to subsequent increases in appetite).

BMI is an imperfect measure of adiposity, especially in children. However, we focused on BMI as it was measured at multiple ages in each of the cohorts. Different chips were used to genotype members of each cohort, though the procedures used to QC and impute genetic data were harmonised across cohorts and we subsetted to a common set of SNPs. Finally, we restricted our analyses to participants of European ancestry to maintain consistency across our cohort samples (sample sizes for non-European ancestry participants would be small in any case); future work is required on more diverse cohorts to examine whether results generalise to the broader population.

## Conclusions

Similar genetic variation, as proxied by polygenic indices, appears to have had more pronounced consequences for BMI in cohorts born later in the obesity epidemic. Genetic associations were stronger at the highest BMI centiles – the part of the distribution that has changed most in recent decades. Findings suggest that the effects of genes on BMI are, to some extent, modifiable. Future research should identify aspects of the environment that can temper genetic predisposition.

## Supporting information

S1 File Table A. Number (%) of eligible participants with valid BMI data by cohort-sweep. Table B. Association between childhood PGI and covariates. Table C. Descriptive statistics according to whether the participant was genotyped or was part of eligible sample (singleton of European inferred ancestry [genotyped] or self-reported White ethnicity [otherwise], born in England, Scotland or Wales). Table D. Heritability of BMI and association between adulthood and childhood PGI and BMI by cohort and follow-up. Fig A. Distribution of PGI and rs1558902 FTO variant by cohort. Fig B. Difference in BMI (SD) according to drop-out at a later sweep by cohort, age of BMI measurement, and age at which drop-out was assessed. Fig C. Difference in PGI according to drop-out at a sweep following the collection of DNA by cohort, PGI, and age at which drop-out was assessed. Fig D. Difference in association between PGI and BMI (kg/m^2^) by age and PGI (adulthood or childhood) for specified pair of cohorts. Fig E. Contour plot of difference in association between adulthood PGI and BMI (kg/m^2^) for specified pair of cohorts at specified combination of ages. Fig F. Contour plot of difference in association between childhood PGI and BMI (kg/m^2^) for specified pair of cohorts at specified combination of ages. Fig G. Association between adulthood PGI and BMI (kg/m^2^) by BMI decile, cohort, age of follow-up. Fig H. Association between adulthood PGI and BMI (kg/m^2^) by cohort, age of follow-up, and selected BMI decile. Fig I. Heatmaps of association between adulthood PGI and BMI (kg/m^2^) by BMI decile, cohort, age of follow-up. Fig J. Association between childhood PGI and BMI (kg/m^2^) by BMI decile, cohort, age of follow-up. Fig K. Association between childhood PGI and BMI (kg/m^2^) by cohort, age of follow-up, and selected BMI decile. Fig L. Association between PGI and BMI (kg/m^2^) by cohort, age, PGI (adulthood or childhood) and covariates used. Fig M. Association between PGI and BMI (kg/m^2^) by cohort, age, PGI (adulthood or childhood) and whether inverse probability weighting (IPW) used to account for selection into genotyped sample. Fig N. Association between PGI and BMI (kg/m^2^) by cohort, age, and PGI. Fig O. Relative contribution of adulthood and multi-ancestry PGIs to BMI by cohort and follow-up. Fig P. Association between PGI and childhood BMI by cohort, age, PGI, and measure of BMI (raw [kg/m^2^] or age- and sex- adjusted z-scores).
(DOCX)
